# A cell’s agony of choice: how to cross the Styx? From morphological to molecular approaches to disclose its decision

**DOI:** 10.1007/s10354-018-0652-0

**Published:** 2018-08-23

**Authors:** Wilfried Bursch

**Affiliations:** Institute of Cancer Research, Medical University Vienna, Borschkegasse 8a, 1090 Vienna, Austria

**Keywords:** History, Apoptosis, Autophagy, Ultrastructure, Cell death categories, Geschichte, Apoptose, Autophagie, Ultrastruktur, Zelltodkategorien

## Abstract

The original “apoptosis–necrosis” concept was based on morphology and (patho)physiological conditions of the occurrence of cell death: (1) apoptosis, with nuclear and cytoplasmic condensation/fragmentation prominent, exclusion of autolysis, considered to result from coordinated self-destruction of a cell; (2) necrosis, with cell lysis prominent, caused by violent environmental perturbation leading to collapse of internal homeostasis. This suggestion initiated a controversial discussion within the scientific community and it soon became clear that the “apoptosis–necrosis dichotomy” was not generally applicable. Nowadays, there is sufficient evidence that cells may activate diverse suicide pathways, thereby allowing a flexible response to environmental changes, either physiological or pathological. The present paper commemorates electron microscopic and cytochemical studies on cell death of cultured human mammary carcinoma cells performed by Adi Ellinger, adding a significant contribution to recognize that autophagy can be involved in regulated cell death, thereby challenging the apoptosis–necrosis dichotomy still predominant in the 1990s.

## Zusammenfassung

Das „Apoptose-Nekrose-Konzept“ wurde auf Basis distinkter Morphologien toter Zellen sowie der (patho)physiologischen Bedingungen ihres Auftretens begründet: (1) Apoptose, Kondensation/Fragmentation von Kern und Zytoplasma prominent, Ausschluss von Autolyse; koordinierte Selbstzerstörung der Zelle; (2) Nekrose, Zelllyse prominent; verursacht durch Zusammenbruch der Zellhomöostase nach massiver Zellschädigung. Dieser Vorschlag hatte eine kontroverse Debatte zur Folge und bald wurde deutlich, dass diese „Apoptose-Nekrose-Dichotomie“ nicht generell anwendbar war. Heutzutage ist allgemein anerkannt, dass Zellen mit unterschiedlichen Selbstzerstörungsprogrammen ausgestattet sind; dadurch wird eine flexible Reaktion einer Zelle auf physiologische oder pathologische Signale ermöglicht. Der vorliegende Artikel erinnert an die elektronenmikroskopischen und zytochemischen Untersuchungen von Adi Ellinger an kultivierten humanen Brustkrebszellen. Hiermit leistete er einen wesentlichen Beitrag zu der Erkenntnis, dass, unter bestimmten biologischen Bedingungen, die Autophagie ein Element des regulierten Zelltods ist; damit verbunden die Infragestellung der in den 1990er-Jahren noch vorherrschenden Einteilung von Zelltodkategorien als „Apoptose-Nekrose-Dichotomie“ .

## Cell death, necrosis, apoptosis, and more: historical aspects

The occurrence of cell death under a variety of physiological and pathological conditions in multicellular organisms has been documented many times during the past 180 years [1–10]. For instance, in 1842, Carl Vogt reported on dead cells in skin of obstetrical toads (*Alytes obstricans*) [1]. In 1871, Virchow described the diversity of cell death as “necrosis” and “necrobiosis” [2]. Subsequently, cell death was reported to occur during metamorphosis of invertebrates and lower vertebrates, and during the development of mammals [8, 9]. In adults, cell loss may occur according to physiological demands, e. g., in 1914, Ludwig Gräper, Royal Anatomy Breslau, published *Eine neue Anschauung über physiologische Zellausschaltung* (A new perspective on physiological cell deletion) [3], the morphological features of which exactly corresponded to apoptosis as defined several decades later. In developmental biology, cell death essentially was considered as a “programmed” event [4, 6, 8, 9]. Notably, Schweichel and Merker [11] and Clarke [12] described three morphologically distinct types of cell death in the developing embryo: type I, most likely identical to apoptosis; type II is characterized by involvement of lysosomes and prominent formation of autophagic vacuoles (“autophagic cell death”); type Ill is described as occurring through disintegration of cells into fragments without involvement of the lysosomal system and without marked condensation. In vivo, cell residues undergoing apoptosis (type I) and autophagic cell death (type II) were reported to finally be phagocytosed by neighboring cells. On the other hand, in toxicology and pathology, cell death mainly was regarded as a passive, degenerative phenomenon occurring after severe damage of tissues [2, 5, 6]. It was not before the early 1970s when Farber et al.—based upon a distinct morphology of cell death along with its requirement for protein synthesis—suggested a “suicide” type of cell death in liver, intestine, and other organs induced by cytotoxic anti-cancer drugs [10]. The widespread occurrence and biological relevance of programmed cell death was also advocated by Kerr, Wyllie, and Currie, who in 1972 proposed a new classification of cell deletion into two broad categories: 1. apoptosis (formerly “shrinkage necrosis”), which “appears to play a complementary but opposite role to mitosis in the regulation of animal cell populations. Its morphological features suggest that it is an active, inherently programmed phenomenon, and it has been shown that it can be initiated or inhibited by a variety of environmental stimuli, both physiological and pathological” [5]. According to this proposal “necrosis,” which often was used for all types of cell death, was re-defined and restricted to events caused by violent environmental perturbation leading to collapse of internal homeostasis [5].

The concept of this “apoptosis–necrosis dichotomy” initiated a controversial discussion, but eventually moved apoptosis, and in a broader sense cell death, into the focus of biomedical research. Nowadays, the scientific community has achieved consensus, considering apoptosis as an essential part of life for any multicellular organism [13–16]. Along with these research efforts, morphological and biochemical observations revealed that self-destruction of cells indeed is not confined to apoptosis as originally defined [8, 9, 17–19]. Nowadays, the knowledge on the cell death regulatory network is considered sufficient to switch from morphological to biochemical criteria for classification of cell death [20, 21]. Consequently, the terms “accidental cell death (ACD)” and “regulated cell death (RCD)” have been suggested by the Nomenclature Committee on Cell Death (NCCD) [20, 21].^1^ These publications include detailed recommendations for the use of biochemical and functional criteria for cell death classification. Accordingly, RCD incl. subroutines comprise “caspase-dependent intrinsic apoptosis,” “caspase-dependent extrinsic apoptosis,” “necroptosis,” “parathanatos,” “ferroptosis,” “netosis,” and others; caspase-unrelated variants of RCD include “autophagic cell death” [20, 21].

The present paper aims at recalling electron microscopic (EM) and cytochemical studies performed by Adi Ellinger (A. E.) on human mammary carcinoma cells in the 1990s. At that time, the apoptosis–necrosis dichotomy still dominated the interpretation of morphological and biochemical data on cell death, but A. E. added a significant contribution to recognize that cells may choose among a number of different tracks to cross the Styx.

## From apoptosis morphology to the complexity of regulated cell death pathways

**Fig. 1:**
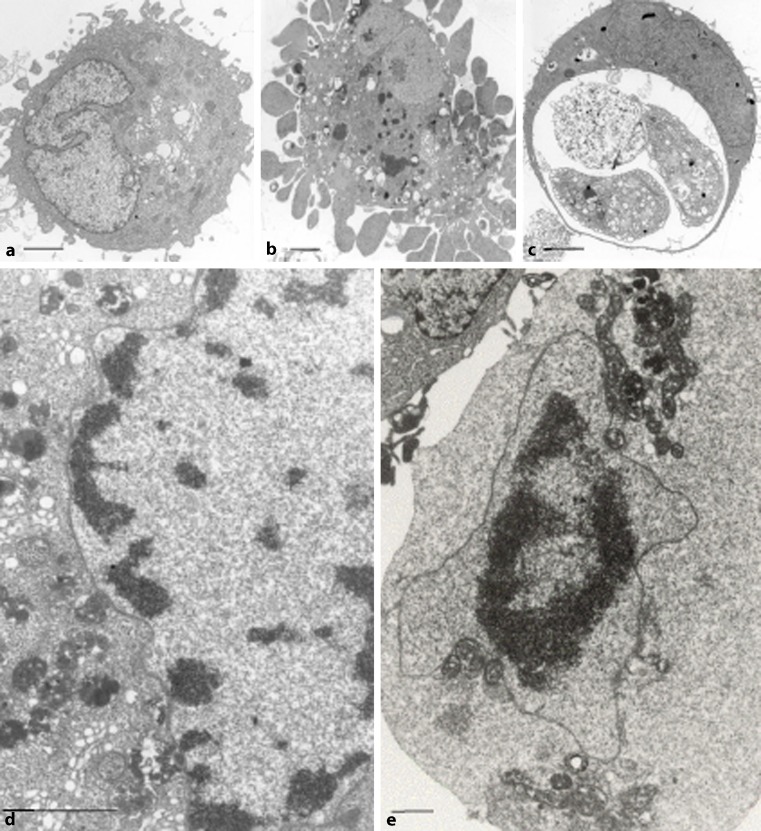
Ultrastructural features of regulated cell death (representative examples). **a**–**c** Human lung carcinoma cells (A549). **a** Control; **b**, **c** 24 h upon 5 µg cisplatin/ml; bars 2 µm. **b** Cell fragmentation into apoptotic bodies; condensed nuclear fragments, adjacent to nuclear envelope. **c** Phagocytosed ABs (apoptotic body), note various stages of degradation (“secondary necrosis”). **d**, **e** MCF-7/7.0.3 cells upon 10-6 M tamoxifen, day 7. **d** Ribbons of condensed chromatin detached from nuclear envelope, note abundant presence of autophagic vacuoles; bar 2 µm. **e** Rounded cell with pyknotic nucleus, amorphous cytoplasm with clustered mitochondria and autophagic vacuoles; bar 1 µm. For experimental details see [30]

The definition of apoptosis was based on cell morphology [5] and includes the following “classical” features: condensation of cytoplasm, in solid tissues separation from neighboring cells, condensation/fragmentation of chromatin at the nuclear membrane to sharply delineated masses (sometimes like crescents), disintegration of cell “into a number of membrane-bound, ultra-structurally well-preserved fragments” (Fig. 1a–c; [5]). In respect of the discussion on autophagy, it should be emphasized that Kerr, Wyllie, and Currie stated that “the evidence suggests that lysosomes are not involved in the genesis of this degeneration” [5]. Likewise, our in vivo cytochemical studies revealed no evidence for autolysis in early (i. e., as long as extracellular) stages of hepatocellular apoptosis [22]. Apoptotic bodies usually are readily phagocytosed and degraded by neighboring cells (Fig. 1c). In vivo, the histologically visible stages of hepatocellular apoptosis were found to last about 3 h [23].

At the Institute of Cancer Research, Vienna, the research interests of the Toxicology unit were focused on chemical carcinogenesis. In addition to in vivo models, we used a number of cell culture models to analyze the mechanisms of action of chemicals as well as endogenous factors (hormones) on the regulatory network of cell proliferation and cell death. In this context, we performed a series of experiments with estrogen receptor-positive human mammary carcinoma cells (MCF-7), a widely used biological model in research on endocrine cancer and drug development [24, 25]. In view of the debate on RCD subtypes, namely autophagic cell death, it is important to note that the “classical” MCF-7 cells turned out to lack functional caspase-3 (MCF-7/7.0.3 cells; 47 bp deletion in exon 3 of the caspase-3 gene) [26–28]. An MCF-7 cell subline reconstituted with caspase-3 was provided in 1998 by Jänicke et al. [24; MCF-7/7.3.28]. To date, accumulating evidence suggests that caspase-3 abundance determines the eventual morphological and biochemical phenotype of cell death [28]. Likewise, bifurcation between apoptosis and necroptosis was found to be dictated by caspase-8 [29].

We and others have used MCF-7/7.0.3 cells as a model to study the anti-survival effect of anti-estrogens such as tamoxifen, ICI 164384, and toremifene [25, 30]. At high concentration, tamoxifen (10-5 M) caused lysis (necrosis) of almost all cells within 24 h that cannot be prevented by estradiol [30]. The cytotoxic action of tamoxifen may result, for instance, from oxidative stress causing a high oxidation status of proteins and DNA [25, 31]. On the other hand, lower concentrations of tamoxifen (10-6 M and below) induced a gradual, dose-dependent appearance of cell death starting to occur approximately 2–3 days after treatment [30]. This type of cell death was considered to be receptor-mediated because of its inhibition by estradiol [30]. Thus, the functional criteria as observed in our studies on MCF-7/7.03, namely (a) cell lysis not prevented by estradiol upon the necrogenic concentration of tamoxifen (10-5M), but (b) pharmacological inhibition of cell death by estradiol upon 10-6 M and below, both of which meet with the most recent recommendations to functionally differentiate (such as pharmacological inhibition) between regulated and accidental cell death [20, 21].

However, in view of the apoptosis–necrosis dichotomy, the antiestrogen-mediated regulated death of MCF-7/7.0.3 cells somewhat surprisingly did not meet completely with the “classical” apoptotic morphotype. Therefore, in cooperation with Adi Ellinger, we studied the dying MCF-7/7.03 cells in more detail at the electron microscopic level. The EM approach revealed two distinct patterns of changes in the nuclei of MCF-7 cells: (a) ribbons of condensed chromatin detached from the nuclear envelope (Fig. 1d), and condensed chromatin to a single, pyknotic mass in the center of the nucleus, detached from the nuclear envelope (Fig. 1e); (b) apoptosis-like condensation and fragmentation of chromatin to crescent masses abutting to the nuclear envelope [30]. Quantitative evaluation at the light microscopic level revealed the predominance of the pyknotic type of nuclear alterations to be three times more frequent than “classical” apoptotic nuclei [30]. The predominant morphological manifestation of pyknotic nuclei was supported by the pattern of DNA degradation as demonstrated by the TUNEL^2^ technique as well as PFGE^2^ and CAGE^2^ gel electrophoresis, all indicating that only a relatively small amount of the total DNA was finally degraded into low molecular weight fragments (20 kb and less) [30]. Overall, these observations were in line with the caspase-3-deficiency of MCF-7/7.0.3 cells [32, 33].

Furthermore, Adi Ellinger demonstrated that regulated death of MCF-7/7.0.3 cells upon tamoxifen exposure was associated with autophagic degradation^3^ of cytoplasmic components preceding the nuclear pyknosis (Fig. 1d). Thus, in cells exhibiting a highly condensed (pyknotic) nucleus, structures required for protein synthesis such as polyribosomes, endoplasmic reticulum (ER) and Golgi have disappeared, whereas a few clusters of intact mitochondria persist in close vicinity to autophagic vacuoles and the nuclear envelope (Fig. 1e; [30]). The electron microscopy studies were confirmed and extended by histochemical studies with monodansylcadaverine (MDC), which has been described to accumulate in autophagic vacuoles (AV) [35]. MDC was used to visualize AVs in MCF-7/7.0.3 cells and to compare the kinetics of AV formation with those of nuclear condensation at the light (fluorescence) microscopy level: AV formation preceded nuclear collapse [30, 36]. Further studies revealed preservation of cytoskeletal elements until late stages [36]; these are known to be necessary for the autophagic process to ensue [34]. Later, Petrovski et al. [37] and Fazi et al. [38] confirmed the kinetics of the occurrence of autophagic vacuoles by microtubule-associated protein 1 *l*ight *c*hain *3* (LC3) expression, along with quantification of nuclear collapse.

Importantly, Fazi et al. [38] performed a comparative approach with MCF-7-7/7.03 lacking functional caspase-3 as well as caspase-3-reconstituted MCF7/7.3.28; cell death was induced by 4-hydroxy(phenyl)retinamide (4-HPR), a synthetic derivative of retinoic acid. 4-HPR-induced death of MCF-7/7.03 cells exhibited histochemical and molecular features of autophagy (increase in autophagosomes, increase in beclin 1 expression, conversion of the soluble form of LC3 to the autophagic vesicle-associated form LC3-II, shift from diffuse to punctate LC3 staining). By contrast, using the same histochemical and molecular criteria, MCF-7/7.3.28 cells with reconstituted caspase-3 exhibited the apoptotic phenotype. These observations showed that 4-HPR may trigger two alternative suicide programs available in MCF-7 cells, one of which associated with autophagy, most probably because of a deregulated apoptotic pathway consequently to caspase-3 deficiency.

Taken together, these observations suggested a certain level of specificity and a feedback mechanism allowing an interaction between autophagic degradation and coordinated completion of the overall cell death process. Furthermore, the immunochemical and biochemical features (beclin1, LC3/LC3-II) found in the caspase-3-deficient MCF-7 cells used in our experiments meet—at least as far as investigated—with currently suggested biochemical criteria for autophagic cell death [20, 21].

However, we always considered morphological features as insufficient to imply a causative relationship between autophagocytosis and eventual manifestation of a cell’s suicide (e. g., [39, 40]).^4^ A crucial question to be answered is whether autophagy might just be a side effect of the stress imposed upon the cells by death stimuli or whether a functional link exists between autophagocytosis and execution of the eventual death program.

In our MCF-7/7.03 model we used 3‑methyladenine (3-MA), at that time a widely applied compound for pharmacological inhibition of autophagy via block of phosphatidylinositol 3‑kinase (PI-3K) activity [41, 42]. 3‑MA indeed inhibited tamoxifen-induced nuclear condensation/fragmentation [30]. These results were confirmed and extended by Petrovsky et al. [37], showing that on day 4 under tamoxifen, more than 95% of the dying cells were MDC positive, and that addition of 3‑MA at that stage almost completely abolished the MDC-positive staining and, as demonstrated by FACS (*f*luorescence *a*ctivated *c*ell *s*orting) analysis, followed by a significant drop in the number of annexin-V-positive and annexin-V-positive/PI(propidium iodide)-positive cells.

Later on, however, it turned out that the action of 3‑MA is not limited to class III PI-3K, but was found to affect multiple targets involved in cell death signaling: class I PI-3K, jun N‑terminal kinase, p38 kinases, mitochondrial permeability transition pore opening [42–44]. The 3‑MA concentration required for effective inhibition of autophagy was also considered to be very high [42]. As to the role of p38 in controlling the balance between apoptosis and autophagy, it is noteworthy that suppression of p38 signaling was recently found to promote necroptotic and autophagic cell death in TNF-alpha-treated L292 fibroblasts [45].

Taken together, according to most recent suggestions by NCCD to attribute RCD to the autophagic subtype, the 3‑MA inhibition experiments performed with our MCF-7/7.03/tamoxifen model do not provide sufficient data to definitively differentiate between RCD *with* autophagy and RCD *by* (i. e., causative relationship) autophagy. For this purpose, pharmacological or genetic inhibition of at least two distinct molecular targets along autophagy signaling should be provided to establish a functional link [21]; the recently described “autosis” may serve as an example [46]. However, data obtained with our MCF-7 model provide sufficient evidence that elements of autophagic and apoptotic pathways may be activated alternatively for self-destruction of MCF-7 cells; the eventual phenotype appears to depend on the absence or presence of functional caspase-3. This is in line with current knowledge on crosstalk between apoptosis and autophagy signaling pathways, as caspase-3 cleaves molecules involved in block of autophagy (e. g., beclin1, Atg4D) [29, 46, 47]. Likewise, the decision between apoptosis and necroptosis was reported to be dictated by caspase-8 [29]. Shimizu et al. [48] provided evidence for an even more complex setting, namely two distinct RCD pathways, but both involving autophagy: (a) in apoptosis-resistant mouse embryo fibroblasts with double knockout of the pro-apoptotic Bax-Bak, cell death depends on autophagy proteins, subjected to control via jun N‑terminal kinase (JNK); (b) during starvation-induced RCD, autophagic vacuoles occur but eventual cell death ensues independently of autophagy proteins.

The progress in biochemical and molecular techniques paved the way for a tremendous increase in knowledge on the molecular biology of cell death. Consequently, NCCD recently recommended switching from morphological to biochemical criteria for classification of cell death [20, 21]. Reviewing the literature on autophagic cell death for compliance with the stringent NCCD criteria suggested that this RCD subtype occurs predominantly in developmental settings, but less frequently or even rarely in mammalian systems [49–54]. In addition to these NCCD criteria, however, most recently the need for a closer look at the autophagic flux has been emphasized, based upon its varying levels in different tissues [55]. These authors highlight the lack of criteria for the amount of autophagy induction necessary to achieve death, because a threshold dividing lethal and protective autophagy appears likely.

To summarize, there is sufficient evidence for a crosstalk between apoptosis and autophagy signaling. Cells are equipped with a number of death pathways which, like a set of building blocks, may be composed to allow a high degree of flexibility in response to death stimuli, either physiological or pathological. Thus, the eventual RCD phenotype most likely appears to depend on general biological settings such as developmental stage, epigenetic and genetic status, metabolic state, and death stimulus.

Finally, in view of the recent progress in molecular biology of accidental and regulated cell death, the question may be raised: are morphological approaches nowadays obsolete? The answer to this question, in the first instance, may be based upon on the limitations inherent to data obtained from whole organ/cell homogenates/extracts. Such limitations arise from the lack of insights specifically into dying cells with respect to function/interaction of distinct organelle(s), spatial distribution of the molecular target under study, and others. The validity of such types of data can be strongly enhanced by histological and cytochemical techniques. Notably, morpho-functional studies of complex and dynamic cell compartments could benefit from recent improvements in preparation procedures for electron microscopy, namely the high-pressure freezing technique, as exemplified by studies on the endocytic compartment performed at the Center for Anatomy and Cell Biology, Medical University Vienna [56, 57]. Thus, the answer to the above question should be “no”. Last but not least, histological and cytochemical techniques are indispensable elements in toxicological pathology, e. g., to evaluate morphologically visible alterations inclunding cell death in organs/cell cultures within the legal framework of chemical safety assessment [58].

## Conclusion

Multiple evolutionarily conserved suicide pathways are available in higher eukaryotic cells; ancient molecular cell death mechanisms have been improved by acquiring complex sets of interacting “death” and “survival” molecules that allow a higher eukaryotic cell to finely tune its life–death decision. Early observations on the diversity of cell death phenomena were mainly based upon morphology, associated with its inherent limitations to establishing causative relationships among subcellular processes. The progress in biochemical and molecular techniques resulted in a tremendous gain in knowledge on the regulatory network of cell death and made functional and molecular criteria for its refined classification available. Consequently, to date, experimental approaches to tackle pending issues can be designed more target oriented. For instance, as to cancer metabolism, the relationship between the rate of autophagosomal protein degradation (autophagic flux) in cancer cells and their susceptibility to the death trigger may deserve attention. In this context, fine structural preservation along with high temporal resolution constitute key elements to elucidate the dynamics of the interaction between cellular compartments involved in cell death signaling and execution. A challenging task for biomedical research will be to understand epigenetic control of cell death. For the time being, I would like to come back to the specific aim of my present contribution, namely to commemorate Adi Ellinger’s contribution to cell death research. A. E., based upon electron microscopy and cytochemistry, provided well-founded arguments to challenge the apoptosis–necrosis dichotomy that was still predominant in the 1990s, eventually leading to a broader view of cell death phenomena.

## Footnotes

1 In the present paper, the recently suggested terms ACD, RCD incl. subroutines [20, 21] are used when justified, the check for their applicability to our observations on MCF-7 cells made in the 1990s is described in the text.

2 **TUNEL:** *T*erminal deoxynucleotidyl transferase (TdT) d*U*TP *n*ick-*e*nd *l*abelling (TUNEL) assay; based on the ability of TdT to label ends of double-stranded DNA breaks independent of a template; designed to detect cells subjected to DNA degradation. **PFGE:** *p*ulsed-*f*ield agarose *g*el *e*lectrophoresis, used for detection of high molecular weight (HMW) DNA fragments (50 kB and above); **CAGE:** *c*onventional *a*garose *g*el *e*lectrophoresis, used for detection of low molecular weight (LMW) DNA fragments (20 kB and below); see [30] for details.

3 Macroautophagy, for review see [34], herein—for the sake of simplicity—referred to as “autophagy.”

4 Historically, denoting cell death as autophagic cell death was based upon electron microscopic demonstration of autophagic vacuoles in dying cells. As stated previously [39], referring to the morphological/cytochemical features should not imply a causative relationship between autophagocytosis and eventual manifestation of a cell’s suicide; this will require either an established functional link between these phenomena and/or elucidation of specifically related genetic/epigenetic events. Our statement was confirmed and extended by the recent NCCD criteria, specifically asking for pharmacological/genetic modulation of at least two targets along autophagy signalling [20, 21].
